# The Role of BDNF-TrkB Signaling in the Pathogenesis of PTSD

**DOI:** 10.4172/2167-1044.S4-006

**Published:** 2013-10-04

**Authors:** Christopher R. Green, Stefani Corsi-Travali, Alexander Neumeister

**Affiliations:** 1 Molecular Imaging Program, Department of Psychiatry and Radiology, New York University School of Medicine, New York, USA; 2 Steven and Alexandra Cohen Veterans Center for the Study of Posttraumatic Stress and Traumatic Brain Injury, Department of Psychiatry, New York University Langone Medical Center, New York, USA

**Keywords:** Posttraumatic stress disorder, Pathogenesis, Neurotrophic processes, Bdnf-TrkB Signaling

## Abstract

Posttraumatic Stress Disorder (PTSD) is a prevalent, chronic, and disabling anxiety disorder that may develop following exposure to a traumatic event. The majority of individuals with PTSD often have comorbid psychiatric conditions such as major depression, generalized anxiety disorder, and substance use disorders, and are at increased risk for suicide. Despite the public health significance of PTSD, relatively little is known about the etiology or pathophysiology of this disorder, and pharmacotherapy development to date has been largely opportunistic instead of mechanism-based. One promising target for modulation is Tropomyosin Receptor Kinase B (TrkB), the receptor for Brain-Derived Neurotrophic Factor (BDNF), a signaling pathway important for neuronal plasticity, survival, and growth. The following discusses how genetic and environmental alterations to this signaling pathway may contribute to anatomical and functional changes in the hippocampus, amygdala, anterior cingulate cortex, ventromedial prefrontal cortex, and the nucleus accumbens. Changes in these brain regions may in turn contribute to the predisposition to or maintenance of some of the clinical manifestations of PTSD, including intrusive memories, hyperarousal, increased fear, and emotional numbing.

## Introduction

**Figure 1:**
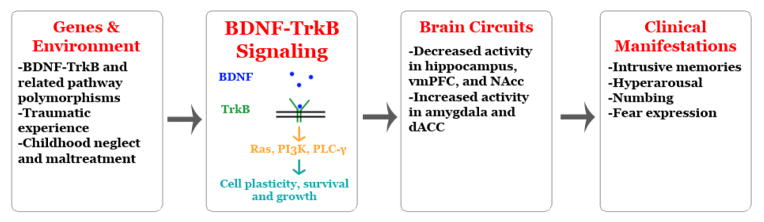
Genetic differences, such as the BDNF Val66Met allele, in combination with environmental exposure, such as childhood neglect, alter the level of BDNF-TrkB signaling, which is important for neuronal plasticity and growth. This contributes to changes in the hippocampus and poorly contextualized fear memories, in the vmPFC and deficient fear extinction, in the NAcc and reduced reward responsiveness, and in the amygdala and dACC, leading to heightened fear response.

Post-Traumatic Stress Disorder (PTSD) is a disease which affects millions of people a year, yet no strong, universal prevention or treatment strategy is available. One promising target for the development of trsategies of treatment and secondary prevention is tropomyosin receptor kinase B (TrkB), the receptor for brain-derived neurotrophic factor (BDNF). This signaling pathway has been implicated in a wide variety of psychiatric diseases, with significant changes in levels of BDNF and TrkB mRNA and protein levels in the hippocampal and pre-frontal cortical regions in the post-mortem brains of individuals with various psychiatric diseases [1–4]. More specific to PTSD, changes in this pathway have been shown to affect contextual fear learning, extinction, and expression and reward responsiveness [5–7]. These alterations may be partially responsible for the development of clinical hallmarks of PTSD such as intrusive memories, hyperarousal, fear, and restricted range of affect (Figure 1). This paper reviews our current understanding of the pathophysiology underlying PTSD with evidence suggesting functional etiologies in the TrkB-BDNF system. By revealing the neurobiological substrates and systems that play a role in the etiology of PTSD, we aim to identify novel targets that offer potential therapeutic value in developing future evidence-based PTSD pharmacologic interventions.

## Search Methodology

Searches were carried out using primarily Google Scholar and NCBI’s PubMed databases. Key search terms used, some in combination, include: “PTSD,” “BDNF,” “TrkB” “neurogenesis,” “stress,” “corticosterone,” “post-mortem,” “depression,” “hippocampus,” “nucleus accumbens,” “prefrontal cortex,” “anterior cingulate cortex,” “amygdala,” “fMRI,” “volume,” “heterozygous,” “child,” “Val66Met,” “5-HTTLPR,” “fear,” “contextual fear,” and “reward responsiveness.” Additional literature was found using review papers to fill in any gaps generated from the above search criteria. The paper itself attempts to describe the strength and quality of evidence found in any given paper, for example, whether it is cross-sectional, genetic, meta-analytic, prospective, or experimental, and whether it was done in humans or animal models. An effort is made to include all literature directly relevant to the arguments made herein, with a special focus on the highest-quality evidence.

## Molecular Biology of BDNF-TrkB Signaling

The effects of BDNF-TrkB signaling are wide ranging and include increased cell plasticity, survival, and growth. TrkB signals through Ras, PI3K, and PLC-gamma and is important for long-term potentiation in the hippocampus [8,9], hippocampal memory consolidation [10], pre-synaptic vesicle docking and release [11,12], increased arborization and synapse number [13], survival of neurons [14], neurogenesis [15], and morphology of neurons [16]. Given its widespread distribution throughout the brain and its broad functional role, the BDNF-TrkB pathway interacts with many other signaling systems relevant to psychopathology, including the serotonin, endocannabinoids, and glutamatergic pathways [6]. Regulation of BDNF-TrkB signaling is multivariate and has not been fully elucidated. However, neuronal activity, positive and negative autocrine and paracrine feedback, gluccocorticoids, and complex endosomal signaling are implicated [17].

## BDNF-TrkB Signaling Affects Brain Circuits Important in PTSD

To understand how PTSD develops, it must be explained how genetic and environmental variables lead to predisposing neurocircuitry abnormalities, which when confronted with a traumatic stimulus, induce or uncover other abnormalities, resulting in sustained PTSD symptoms. Using various prospective, twin, and genetic studies, there has been some recent work that attempts to differentiate which abnormalities present in individuals with PTSD pre-date the traumatic experience from those acquired during and after the trauma [18]. Building on this work, the following explains the importance of BDNF-TrkB signaling in the hippocampus, amygdala, anterior cingulate cortex (ACC), ventromedial prefrontal cortex (vmPFC), and nucleus accumbens (NAcc) in the pre-disposition to and maintenance of PTSD symptoms. These structures are highly interconnected and together help determine behaviors important in PTSD [19].

## Hippocampus

Cross-sectional studies have associated decreased hippocampal volume, especially in the cornu ammonis region 3 and the dentate gyrus of the hippocampus, with PTSD [20,21]. Stronger evidence for decreased hippocampal volume as a true predisposing factor for PTSD comes from monozygotic twin studies [22,23], although decreases in hippocampal volume may also be acquired post-trauma [24,25]. Functionally, declarative memory deficits have been identified in humans with PTSD [26,27] and animal lesion studies have found the hippocampus to be vital for developing contextual fear memory [28]. In addition, blocking neurogenesis in the hippocampus has been shown to reduce contextual fear learning [29]. Thus, impaired contextual encoding offers one possible explanation for re-experiencing of aversive memories in inappropriate contexts.

While childhood neglect and maltreatment clearly pre-dispose individuals towards increased amygdala reactivity, it is more uncertain whether these factors contribute to decreased hippocampal volume. Some have found decreased hippocampal volume among those maltreated as children [30], while others have found no effect among people who were institutionalized or had depressed mothers as children [31,32]. Although the effect of childhood environment is uncertain, animal models have found that stress and exogenous corticosterone alter BDNF and TrkB mRNA and protein levels in the hippocampus [33–36], pre-natal and adult stress decrease neurogenesis in the hippocampus [37–41], and stress and exogenous corticosterone decrease hippocampal volume [42,43].

In addition to potential stress induced changes in BDNF-TrkB signaling and hippocampal volume, genetic variation in this pathway probably also contributes to some PTSD vulnerability with respect to hippocampal volume. The BDNF Met allele is associated with declarative memory deficits, decreased hippocampal NAA levels [44], and decreased hippocampal volume [45–49], although others have found no effect on volume [50–52]. In addition, BDNF signaling is vital for neurogenesis in the hippocampus [15,53]. As was discussed earlier, BDNF-TrkB signaling has been found to be important for long-term potentiation in the hippocampus [8], hippocampal memory consolidation [10], increased pre-synaptic vesicle docking and release in hippocampal neurons [11,12], and increased arborization, synapse number [13], survival, [14] and normal morphology of neurons [16]. Most importantly, animal models of BDNF heterozygous knockouts and TrkB heterozygous knockouts show diminished hippocampal-LTP, with corresponding decreases in contextual fear conditioning [5,9,54,55]. Furthermore, overexpression of TrkB in mice led to improved memory, contextual fear conditioning, and overall reduced anxiety [56]. Finally, BDNF expression in the hippocampus is vital for extinction of fear memories [57]. These findings suggest that a genetic and/or environmentally caused deficiency in the BDNF-TrkB signaling pathway could lead to improper development and maintenance of hippocampal volume, robustness, and plasticity. This could predispose individuals to poorly contextualized memories and weakened fear extinction, ultimately contributing to intrusive memories and difficulty forming new memories.

## Amygdala and anterior cingulate cortex (ACC)

Given the importance of both of these structures in bringing about the fear response, and in the case of the dACC inhibiting fear extinction, increased responsivity to emotional stimuli in these areas may account for some of the increased fear expression, hyperarousal and to a lesser extent intrusive memories seen in PTSD [18,58–60]. In two prospective studies of military personnel, increased activity in the amygdala in response to emotional stimuli was associated with a greater vulnerability to developing PTSD symptoms [24,61]. Increased resting metabolic activity and activation to an interference task in the dorsal anterior cingulate cortex (dACC) also predicts an increased likelihood of developing PTSD [62,63]. In addition to changes in dACC activity, overall decreases in ACC volume are associated with a predisposition to develop PTSD among adolescents and adults [64,65]. Furthermore, the BDNF Val66Met polymorhism, which results in decreased activity dependent secretion of BDNF [44], has been associated with decreased ACC grey matter volume (GMV) among adolescents [66]. This suggests a potential role for BDNF-TrkB signaling in this area. Deficient BDNF secretion could lead to inadequate growth signaling in the ACC, diminished volume in this region, and ultimately a predisposition to developing PTSD. Whether and how overall changes in ACC GMV are related to dACC activity specifically is unclear.

Changes in the function of the amygdala have been associated with childhood maltreatment, deprivation, and institutionalization [31,67–70] and genetic polymorphisms, such as the serotonin transporter (5-HTT) gene [71]. BDNF may influence the serotonin system as it relates to amygdala activity. The serotonin transporter linked polymorphic region (5-HTTLPR) short allele is associated with increased amygdala activation in response to emotional stimuli [72] and with PTSD among individuals with childhood adversity [73]. Furthermore, in the setting of depression, evidence indicates that, among maltreated children, Val66Met alleles in combination with 5-HTTLPR short alleles produces the greatest risk for depression [74,75]. This suggests that BDNF signaling could also play a role in modulating the effect of the serotonin system on amygdala dysfunction in PTSD.

Nonetheless, it should be noted that increased BDNF signaling could theoretically increase the risk of PTSD, if present in certain brain areas such as the amygdala. During fear acquisition BDNF expression and TrkB phosphorylation are increased in the basolateral amygdala, while inhibition of TrkB in the BLA resulted in disrupted fear acquisition [76]. All together, the above suggests that dysregulated BDNF signaling may interfere with normal functioning of the ACC and amygdala, leading to heightened fear responses, ultimately contributing to the potential development of PTSD.

## Ventromedial prefrontal cortex (vmPFC)

Another major brain area important in PTSD is the ventromedial prefrontal cortex, an area involved in fear extinction [6]. In the lab, humans who have acquired PTSD have increased fear acquisition, yet decreased fear extinction [77–79]. Some evidence does suggest that the fear extinction deficit in PTSD is an acquired trait rather than a pre-existing one [80]. Nonetheless, even if impaired fear extinction is more of an acquired deficit, it may contribute to the maintenance of many clinically relevant features of PTSD, such as re-experiencing, avoidance, and anxious arousal. Functionally, changes in fear extinction among those with PTSD have been associated with under-activation of the vmPFC and over-activation of the dACC during extinction training [81]. The vmPFC is thought to generally exert inhibitory control over the amygdala and is important for acquiring and recalling fear extinction [58,82–84]. Moreover, BDNF signaling likely plays an important role in this brain region. BDNF met alleles have been associated with deficient fear extinction and activation in the vmPFC, [85,86] the process of fear extinction increases BDNF in the vmPFC, [87] and systemic BDNF agonist increases fear extinction [88].

## Nucleus accumbens (NAcc)

Another brain region implicated in the pathogenesis of PTSD is the nucleus accumbens area. Evidence indicates that in PTSD the nucleus accumbens may be less active, corresponding to a decrease in reward responsiveness [89,90]. However, one prospective study suggests that decreased NAcc responsivity may be an acquired, post-traumatic deficit [61]. Nonetheless, individuals with decreased BDNF-TrkB signaling may be less able to cope with diminished activity in the NAcc due to a combination of genetic vulnerability and traumatic experience. Indeed, both BDNF and TrkB expression are vital for reward responsiveness in BDNF knockout and replacement models in mice [91,92]. In humans, there is some evidence that the BDNF met allele diminishes reward seeking and aversive stimuli avoidance behavior [7]. In conclusion, diminished BDNF signaling in the NAcc may contribute to decreased reward responsiveness, leading to the restricted range of affect, or emotional numbing, commonly seen in individuals with PTSD.

## Peripheral BDNF and PTSD

Changes in peripheral concentrations of BDNF have been found in a diverse set of psychiatric diseases, including schizophrenia, depression, bipolar disorder [93–95]. Peripheral measurements of BDNF, in addition to more central ones such as measurements from CSF, provide a potential way to both study the pathogenesis of PTSD as well as act as a biomarker of disease. Evidence for the relevance of peripheral BDNF comes from an experiment that administered exogenous BDNF peripherally in an animal model. BDNF was found to improve anxiety and depression characteristics across different tasks. On a cellular level, exogenous peripheral BDNF increased neurogenesis in the hippocampus, with elevated hippocampal BDNF, pCREB, and Perk [96]. This suggests that BDNF has the ability to cross the blood brain barrier and that peripheral BDNF may potentially be relevant for studying psychiatric diseases.

Overall, there is moderately strong evidence to suggest that increased peripheral BDNF is associated with a current diagnosis of PTSD. One study found increased BDNF serum concentrations among those with PTSD, but only in the PTSD group whose traumatic experienced occurred in the past year [97]. Another paper found increased serum BDNF among those with un-medicated PTSD, with a corresponding positive correlation between Clinician-Administered PTSD Scale (CAPS) score and serum BDNF level [98]. Among those with the BDNF Val66Met allele, those with a probable diagnosis of PTSD had higher average BDNF plasma concentrations [99]. Nonetheless, one study did find a conflicting result, in that those with PTSD had lower plasma BDNF levels compared to controls [100].

Although there is evidence that suggests an elevation in peripheral BDNF after developing PTSD, it is unclear how this is related to the pathogenesis of the disease. For instance, it may be a compensatory response, rather than a predisposing one. More prospective studies need to be carried out to determine how peripheral BDNF levels are related to the neurocircuitry models discussed in the other sections of this paper.

## Conclusion

Overall, it appears that alterations in BDNF-TrkB signaling may play an important role in PTSD. In the hippocampus, vmPFC, ACC, and NAcc deficient BDNF-TrkB signaling likely aids in the pre-disposition to maintenance of impaired contextual fear learning, fear extinction, and restricted range of affect. In turn, these may be associated with some of the clinical manifestations of PTSD, such as intrusive and incomplete memories, hyperarousal, fear expression, and restricted range of affect.

Although a decrease in signaling in the BDNF-TrkB pathway is likely a contributing factor in PTSD, as a neurotrophin and regulator of plasticity, changes in either direction in BDNF signaling, depending on its location, intensity, and time course may moderate the risk of developing stress related psychopathology. In animal models, systemic administration of TrkB receptor modulators have resulted in changes in phenotypes related to human PTSD, such as fear acquisition and extinction [88] and anxiety related phenotypes [101]. These experiments suggest a potential therapeutic role for altering BDNF-TrkB signaling. More translational research in humans in now needed to verify and refine animal models. For example, PET imaging studies using radioligands specific for TrkB may be used to help identify in vivo changes in BDNF-TrkB signaling in various brain regions. Although a drug targeting this system for treatment of PTSD in humans is currently unavailable, research into this area may someday provide a means of preventing or treating patients with PTSD.
